# Complete cleft of the upper limb: A very rare anomaly

**DOI:** 10.4103/0970-0358.44946

**Published:** 2008

**Authors:** Edward T. Bersu

**Affiliations:** Department of Anatomy, University of Wisconsin School of Medicine and Public Health, Madison, Wisconsin 53706

The authors of the manuscript state that the complete cleft of the upper limb which they describe has not been previously reported in the literature. I certainly concur with this statement and have not heard or seen of any case that comes close to their description. The issue is a bit further compounded, in my opinion, by the duplication of the thumb.

This case report certainly deserves to be published and the results disseminated widely. The descriptions of the anatomical and clinical features are concise, as are the results from the imaging studies that were made. I reviewed the figures accompanying the manuscript and they certainly helped to verify the written descriptions. I am an anatomist who has been involved in studies of multiple congenital malformation syndromes. Therefore it would be inappropriate for me to comment on the surgical procedures that have been carried out, although it certainly appeared from the photographs that surgery was successful.

The discussion for the case is very brief and consists of a very general comparison of the reported case with the cluster of defects categorized as “cleft hand” and “mirror hand”. The authors do not offer any developmental basis or pathogenesis mechanism to explain the condition. Given that it is a “first”, I can appreciate that any such thoughts would be highly speculative. I have included, below, parenthetically, my own thoughts about the condition.

In conclusion, I strongly support the publication of this very well written description of a case of a cleft upper limb.

In the case described in this report, one could suggest that, somewhat late in development, the ulnar half of the forearm and related portion of the hand were “split away” from the radial half of the limb, and the two resulting halves “healed”, with the ulnar half being “carried away” a bit from the radial half. The observation that “hand flexors were strong in the inferior limb while the extensors were stronger in the superior limb” suggests a significant amount of “normal” muscle development, where the flexors of the anterior compartment are shifted towards the ulnar half of the limb and the extensors of the posterior compartment are shifted towards the radial half. In addition to the vasculature, it would be instructive to have a picture of the brachial plexus and its branching pattern in the limb.

It is difficult for me to propose a specific pathogenesis mechanism for this cleft of the upper limb using the results of experimental manipulations of such things as the ZPA, apical ectodermal ridge, fibroblast growth factor, etc., although disruptions in these entities certainly must be part of any consideration. I wish to propose a more “gross” possibility. The developing upper limb initially has a single, centrally placed axial artery that becomes the interosseous artery of the forearm. The radial and ulnar arteries “grow out” distally from the brachial artery along their respective sides of the limb, from an origin that can be described as in the region of the developing elbow. Could an early disruption in that centrally placed artery, resulting in a line of necrosis down the central axis of the forearm, lead to a “division” of the radial and ulnar halves?

